# Serum neurofilament light as a predictor of outcome in subarachnoid haemorrhage

**DOI:** 10.1007/s00701-023-05673-9

**Published:** 2023-06-23

**Authors:** Conny Johansson, Helena Aineskog, Lars-Owe D. Koskinen, Andreas Gunnarsson, Peter Lindvall

**Affiliations:** 1 Department of Clinical Science, Neurosciences, Umeå University, SE-901 87, Umea, Sweden; 2 UmanDiagnostics®, Umea, Sweden

**Keywords:** Subarachnoid haemorrhage, Neurofilament protein l, Glasgow Outcome Scale, Cerebral vasospasm

## Abstract

**Background:**

Prognostication of clinical outcome in patients suffering from aneurysmal subarachnoid haemorrhage (SAH) is a challenge. There are no biochemical markers in routine use that can aid in prognostication. Neurofilament light (NFL) measured in cerebrospinal fluid (CSF) has been associated with clinical outcome in previous studies.

**Objective:**

To investigate if serum levels of NFL correlate with CSF levels and long-term clinical outcome in patients suffering from SAH.

**Methods:**

We conducted an observational cohort study of 88 patients treated for SAH at Umeå University Hospital in 2014–2018. Serum and CSF samples were analysed using an enzyme-linked immunosorbent assay to quantify NFL levels. Outcome was assessed using Glasgow Outcome Scale Extended and dichotomised as favourable or unfavourable. Differences in NFL levels between outcome groups were analysed using repeated measurements ANOVA. Relationship between CSF and serum NFL levels was analysed using Pearson’s correlation. A multivariate binary logistic regression model and a receiver operation characteristic curve were used to assess the predictive value of serum NFL.

**Results:**

A significant correlation between serum and CSF-NFL levels could be seen (Pearson’s correlation coefficient = 0.7, *p* < .0001). Mean level of serum NFL was higher in the unfavourable outcome group than the favourable outcome group (*p* < .0001), in all epochs of SAH, and correlated with initial disease severity on the World Federation of Neurosurgical Societies scale. Serum NFL in the late phase displayed the best predictive potential in a receiver operation characteristic curve analysis (AUC=0.845, *p* < .0001).

**Conclusion:**

Levels of NFL in serum and CSF are correlated. Early serum NFL levels seem to reflect initial tissue damage and serum NFL levels in the late phase may reflect secondary events such as vasospasm or delayed cerebral ischemia. Serum NFL may be used as a prognostic marker of clinical outcome in SAH.

**Supplementary Information:**

The online version contains supplementary material available at 10.1007/s00701-023-05673-9.

## Introduction

Aneurysmal subarachnoid haemorrhage (SAH) is a severe form of haemorrhage with high morbidity and mortality [19]. The clinical outcome from SAH varies, from immediate death to return to normal life. Mortality is around 50%, ranging from 8 to 67% [16]. It has been estimated that between 36 and 55% of the patients survive with an independent functional status defined as modified Rankin scale score of 0–3 [15]. The extent of the initial bleed, rebleeding, vasospasm and delayed cerebral ischemia (DCI) have been shown to contribute to outcome [12]. The diversity of the disease makes it difficult, in the acute phase, to predict the long-term outcome. Initial clinical status measured using the World Federation of Neurosurgical Societies (WFNS) scale and aneurysm size, age and amount of blood on computed tomography (CT) measured using the Fisher grade scoring system have been shown to be independent predictors of outcome in SAH [18]. However, these predictors do not take secondary events such as vasospasm or DCI into account. There are no biochemical biomarkers in routine clinical use today that can aid in predicting long-term outcome.

Neurofilament light (NFL) is part of a family of proteins mainly involved in the cytoarchitecture of the neuronal axon. It has been vastly explored as a biomarker of disease activity and severity, mainly in neurodegenerative diseases, but also in stroke and traumatic brain injury [1]. NFL has mainly been studied in cerebrospinal fluid (CSF) since measurement in blood has been difficult. The introduction of highly sensitive analyses has made it possible to study NFL in blood, which facilitates use of this potential biomarker [10]. NFL has been shown to correlate with clinical outcome in serum (sNFL) as well as CSF [3, 6, 13, 22]. Reference values of NFL in blood have been studied in healthy populations [5, 17], showing that age, blood volume and body mass index affect its levels [9].

Hviid et al. showed an association between plasma NFL levels upon admission and poor functional outcome (modified Rankin scale >4) after 30 days in patients suffering from SAH [6]. Association between plasma NFL levels and mortality has been seen in all types of strokes [4]. Longitudinal data with repeated NFL samples from blood in SAH patients have previously only been examined in two studies [3, 22].

The aim of this study was to study how sNFL changes over time in the acute phase of SAH, how this correlates to long-term clinical outcome, and if it could be used as a biomarker for predicting clinical outcome. We also examined the association between CSF-NFL and sNFL levels. Our hypothesis was that sNFL levels would be associated with long-term clinical outcome in patients suffering from SAH and that NFL could be used as a biomarker to aid prognostication.

## Methods

### Study design

This was a prospective observational cohort study. Patients ≥18 years of age who were treated for aneurysmal SAH at Umeå University Hospital in 2014–2018 were eligible for inclusion. Upon arrival, all patients were evaluated by the physician on duty, and classified according to WFNS scale. The patients underwent either a CT angiography (CTA) and/or digital subtraction angiography (DSA) to determine the source of bleeding. This was treated using either endovascular or microsurgical technique based on the judgement of an experienced vascular neurosurgeon in consultation with an endovascular interventionist. The patients were treated in accordance with a local standardised intensive care unit protocol, for at least 10 days, before admission to a rehabilitation unit. If hydrocephalus was present, it was treated with an external ventricular drainage (EVD), and—if persisting—with a ventriculoperitoneal shunt. As prophylaxis for vasospasm, nimodipine (Nimotop®, Bayer, Leverkusen, Germany) 0.2 mg/ml was administered as an intravenous infusion at a rate of 1–15 ml/h for 10 days, followed by peroral treatment of 60 mg six times daily for 21 days, starting upon arrival to the neurosurgical department. Delayed cerebral ischemia was defined as newly arisen neurological deficits that could not be attributed to any other cause (such as hydrocephalus or rebleeding), as determined by the treating clinician. The patients were considered to have vasospasm if this was described by the radiologist on any CTA or DSA conducted during the stay. Venous sampling was performed by a specialised research nurse, starting upon arrival (day 0) and then repeatedly thereafter. Our aim was to collect samples every third day. However, the intervals varied between patients depending on availability of research staff. In addition to venous samples, cerebrospinal fluid (CSF) drawn from an EVD in 20 patients where CSF was available in the early (days 0–3) and late (days 10–14) phase was analysed to correlate with serum samples. Patients were followed up at least 12 months (mean=18, SD=7.5) after ictus through phone interviews conducted by a research nurse. If a patient was unable to communicate, next of kin answered the questions. The patients were scored based on the Glasgow Outcome Scale Extended (GOSE) [7, 20], and results were dichotomised as unfavourable (GOSE 1–4) or favourable (GOSE 5–8). Exclusion criteria were if the source of bleeding was not an aneurysm or if venous samples or follow-up data were missing. Written informed consent was given by each patient, or—if the patient could not consent due to disease severity—by next of kin.

The study was approved by the Regional Ethics Committee (2013/366-31) and followed the ethical standards of the Helsinki declaration.

### Sample preparation and analysis

The venous serum samples were collected in 10-ml glass tubes (BD vacutainer) and then centrifuged, fractionated and frozen at −80 °C within 45 min. Samples were analysed by UmanDiagnostics®, blinded to all clinical data. The UmanDiagnostics NF-light™ Serum enzyme-linked immunosorbent assay (ELISA) is an enzymatic immunoassay designed for quantitative measurements of NFL in human serum. The test uses two highly specific, non-competing, monoclonal antibodies. The capture antibody is coated on a solid surface and binds the sample NFL. The secondary/detection antibody is biotin-conjugated, and addition of horseradish peroxidase-conjugated streptavidin allows for quantitative determinations by enzymatic turnover of a colourless substrate (TMB) to a coloured product. The absorbance value can be correlated to the amount of NFL in the sample, using a calibrator curve. All assay components were allowed to reach room temperature (18–25 °C) before use. Serum and CSF samples were allowed to thaw at room temperature on the benchtop before dilution, in accordance with the protocol. For more detailed information, see supplemental information. Values of NFL are given in pg/ml.

### Statistical analysis

Serum NFL values were divided between early (days 0–2), mid (days 3–6) and late (days 7–18) epochs. If a patient had more than one sample in the same epoch, the highest value was chosen. All analyses were performed using the software SPSS® (IBM®, version 27). The level of significance was set to *p*<.05. All tests were two-tailed. Data variation was analysed using Shapiro-Wilk test and logarithmic transformation was performed to normalise data when deemed necessary. Values are expressed as median ± interquartile range (IQR), mean ± standard deviation (SD) or 95% confidence intervals (CI). Baseline characteristics were compared between groups using the independent samples *t*-test for continuous variables and the chi-squared test for categorical variables. Pearson’s correlation coefficient was calculated to compare CSF and serum levels of NFL. Analysis of covariance (ANCOVA) was performed to compare sNFL levels between treatment modalities. Potential predictors were analysed through a correlation matrix using Spearman’s rho. A repeated measurements mixed ANOVA was conducted to analyse differences in sNFL in all three epochs, followed by a Bonferroni post hoc analysis. Since we did not have a control group, patients were dichotomised as having a high or normal sNFL based on the estimated age-adjusted upper reference intervals suggested by Hviid et al. [5]. A chi-squared test was used to compare the frequency of high sNFL between the groups and these dichotomised sNFL values were used in regression modelling. For regression modelling, a univariate binary logistical regression model using dichotomised GOSE as the dependent variable was used to assess possible predictors. Significant variables were then incorporated into a multivariate analysis. Nagelkerke pseudo *R*^2^ value was used to describe goodness of fit. Lastly, receiver operating characteristic (ROC) curves were created to assess the ability of sNFL to predict outcome.

## Results

### Participants

A total of 147 patients were assessed for inclusion. Four patients did not give consent. No source of bleeding could be found in 18 patients. In two patients, the source of bleeding was not an aneurysm. Venous samples were missing in 32 patients because they arrived at a time when there was no research staff available. Three patients were lost to follow-up. In total, 88 patients were analysed. One patient could not be assessed on the WFNS scale prior to treatment due to being intubated after undergoing oropharyngeal surgery, when a pupil dilation led to the diagnosis of SAH. Baseline characteristics are summarised in Table 1. Twenty-six (30%) patients had an unfavourable outcome and 62 (70%) had a favourable outcome (Table 2). The number of samples collected each day is summarised in the supplemental material. The study population has been described previously by Johansson et al. [8].

**Table 1:** Patient characteristics. Favourable—GOSE 5–8. Unfavourable—GOSE 1–4. One patient in the favourable outcome group could not be clinically assessed prior to treatment. Statistically significant differences are marked with *. The population has been presented previously by Johansson et al. [8]. *GOSE* Glasgow Outcome Scale Extended. *SD* standard deviation. *WFNS* World Federation of Neurosurgical Societies. *DCI* delayed cerebral ischemia. *EVD* external ventricular drainage

Characteristics		Outcome measured as dichotomised GOSE			*p*-value
		Total	Favourable	Unfavourable	
Age (years)	Mean (±SD)	59±13	56±13	66±11	.002*
Hypertension	Yes (%)	43	44	42	.915
Current smoking	Yes (%)	38	40	31	.398
Sex	Female (%)	75	69	89	.059
Multiple aneurysms	Yes (%)	18	13	31	.047*
Aneurysm location	Anterior/posterior (%)	84/16	87/13	77/23	.234
Treatment modality	(%)				.092
	Endovascular	41	39	46	
	Surgical	55	60	42	
	Combined	2	2	4	
	No treatment	2	0	8	
Fisher grade	(%)				.025*
	1	1	1	0	
	2	6	8	0	
	3	24	31	8	
	4	69	60	92	
WFNS scale score	(%)				<.0001*
	1	41	56	8	
	2	20	23	12	
	3	6	7	4	
	4	16	8	35	
	5	17	7	42	
DCI	Yes (%)	10	10	12	.793
Vasospasm	Yes (%)	31	32	27	.621
EVD	Yes (%)	49	37	77	<.0001*

**Table 2:** Outcome 1 year after ictus

GOSE score	Favourable (*n*, %)	Unfavourable (*n*, %)
1		12 (14%)
2		2 (2%)
3		5 (6%)
4		7 (8%)
5	7 (8%)	
6	17 (19%)	
7	15 (17%)	
8	23 (26%)	
Total	62 (70%)	26 (30%)

*GOSE* Glasgow Outcome Scale Extended

### Neurofilament light correlates with initial disease severity and increases over time

**Fig. 1:**
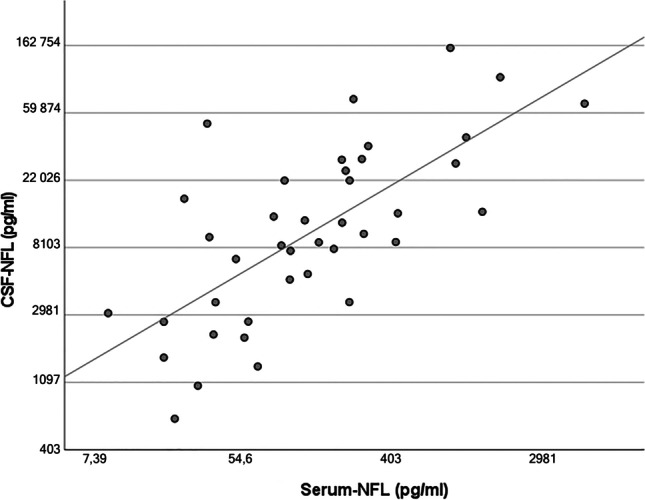
Scatterplot describing the relationship between serum and cerebrospinal fluid (CSF) levels of neurofilament light (NFL) collected at the same time. The *x* and *y* axes have natural logarithmic scales because of non-parametric data distribution. Twenty patients were sampled at two occasions. Pearson’s correlation coefficient = 0.7, *p*<.0001

**Fig. 2:**
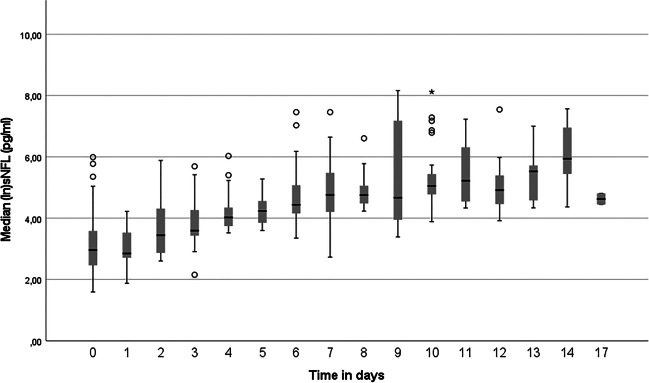
Boxplot presenting the median and interquartile range of serum neurofilament light (sNFL) levels during different days since hospitalisation

**Fig. 3:**
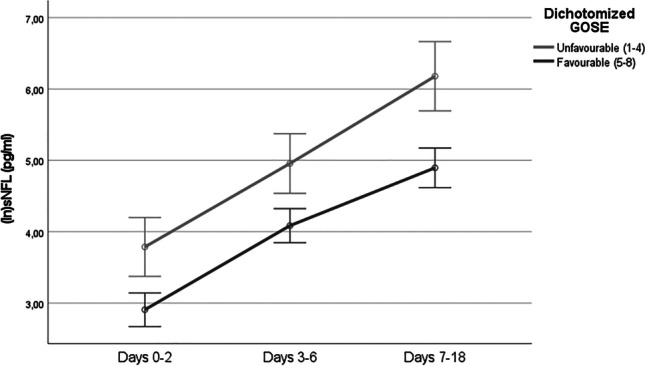
Difference in serum neurofilament light (sNFL) levels between unfavourable and favourable outcome groups at different time epochs, analysed using a repeated measurements mixed ANOVA. Bonferroni-adjusted post hoc tests revealed significant difference (*p*<.0001) between outcome groups in all time epochs. GOSE, Glasgow outcome scale extended. ANOVA, analysis of variance

Levels of sNFL correlated positively with initial disease severity measured clinically as WFNS scale score (rho=0.52, *p*<.0001) and radiologically as Fisher grade (rho=0.27, *p*=.006) (Table 3). There was also a positive correlation between serum and CSF levels of NFL (Pearson’s *r* correlation=0.7, *p*<.0001, Fig. 1). Serum NFL levels seemed to increase over time in the acute phase of SAH, peaking around days 9–14 (Fig. 2). The ANOVA analysis showed significant difference in sNFL levels between epochs, *F*(2, 126) = 184.57, *p*<.0001, partial *η*^2^=0.75 (Fig. 3). No significant differences in sNFL levels in the late phase could be seen between endovascularly and microsurgically treated patients in an ANCOVA, controlling for age and WFNS scale score (*p*=.63).

**Table 3:**
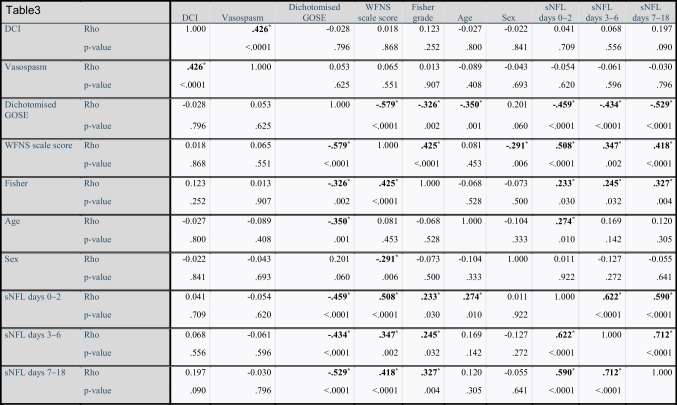
A correlation matrix showing Spearman’s rho rank correlation between different predictors and outcome parameters. *Rho* Spearman’s rho rank correlation coefficient

Significant values are bolded and marked with *. *DCI* delayed cerebral ischemia. *WFNS* World Federation of Neurosurgical Societies. *sNFL* serum neurofilament light. *GOSE* Glasgow Outcome Scale Extended

### Neurofilament light is associated with long-term clinical outcome

The ANOVA showed significant difference between dichotomised GOSE scores, *F*(1, 63) = 21.76, *p*<.0001, *η*^2^=0.26 (Fig. 3). Serum NFL levels showed a negative correlation with dichotomised GOSE, which was strongest in the late phase (Table 3). A larger proportion of the unfavourable outcome group (80%) had sNFL levels higher than the reference value compared with the favourable outcome group (47%) in the early phase (*p*=.005). In the mid phase, only three patients (4%) had normal sNFL levels, and in the late phase, none had normal sNFL levels. Neither DCI nor vasospasm showed significant correlation with sNFL levels at any epoch.

### Predictive value of neurofilament light

**Fig. 4:**
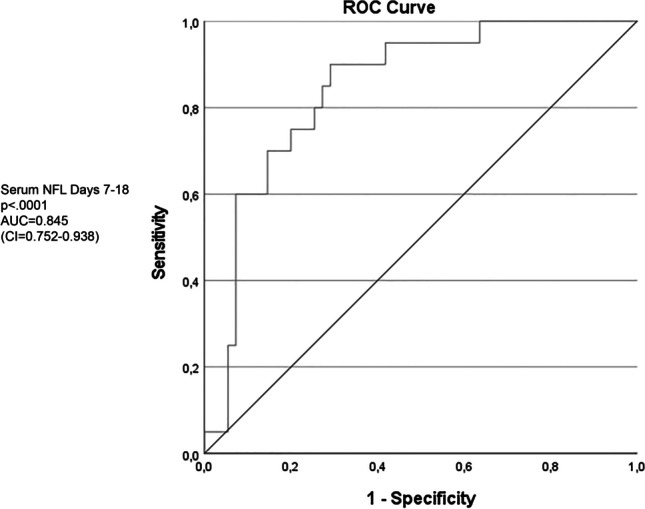
Receiver operating characteristic curve using levels of serum neurofilament light (NFL) for predicting an unfavourable outcome in the late phase (days 7–18 after arrival at hospital). AUC, area under the curve. CI, 95% confidence interval

Since sNFL values differed between outcome groups, ROC curves using sNFL from different epochs were created to assess its value in predicting an unfavourable outcome. The largest area under the curve (AUC) was seen for late-phase sNFL levels (AUC=0.845, CI 0.752–0.938, *p*<.0001, Fig. 4). The ROC curve provides different values of sensitivity and specificity depending on which cut-off value you choose. In the clinical setting, a high specificity is prioritised since the goal is to identify patients with a low chance of favourable outcome. According to our data, setting the cut-off value at 411 pg/ml would yield a sensitivity of 60% and a specificity of 93%. This yields the highest possible sensitivity while keeping specificity over 90%. In a univariate regression model, sNFL on days 7–18 had an odds ratio (OR) of 0.99 (CI 0.099–1.00, *p*=.0012) for predicting a favourable outcome, with a Nagelkerke pseudo *R*^2^ value of 0.151. WFNS scale score alone had an OR of 0.37 (CI 0.25–0.56, *p*<.0001) and a Nagelkerke pseudo *R*^2^ value of 0.45. Fisher grade and age were the other covariates that showed significant effects in univariate analysis (Table 4). In a multivariate regression model using significant variables from the univariate analyses, the Nagelkerke pseudo *R*^2^ increased to 0.592, but in this model, only age and WFNS scale score retained significance (Table 4).

**Table 4:** Univariate and multivariate binary logistic regression analysis for predicting a favourable outcome using dichotomised Glasgow Outcome Scale Extended as the dependent variable

	OR	95% CI	*p*-value
Univariate			
Age	0.94	0.89–0.98	.004*
WFNS scale score	0.37	0.25–0.56	<.001*
Vasospasm	1.29	0.47–3.57	.621
DCI	0.82	0.19–3.57	.793
Fisher grade	0.15	0.03–0.65	.011*
Sex	3.39	0.91–12.67	.07
Premorbid hypertension	1.05	0.42–2.66	.92
Treatment modality	0.78	0.39–1.59	.50
sNFL days 7–18	0.99	0.99-1.00	.012*
Multivariate			
sNFL days 7–18	0.99	0.99–1.00	.17
WFNS scale score	0.40	0.24–0.68	<.001*
Fisher grade	0.29	0.05–1.60	.15
Age	0.90	0.84-0.97	.005*

Statistically significant values are marked with *. *OR* odds ratio. *CI* confidence interval. *WFNS* World Federation of Neurosurgical Societies. *DCI* delayed cerebral ischemia. *sNFL* serum neurofilament light

## Discussion

In the present study, serum and CSF levels of NFL correlated in patients suffering from SAH, which is in accordance with the results of Garland et al. [3]. This important finding indicates reliability when using serum samples for analysing NFL.

The strong correlation between serum and CSF levels of NFL is especially important since only 34% of patients with SAH need an EVD [2]. Previous studies have shown differences in CSF levels of neurofilaments between unfavourable and favourable outcome groups, and our results supported those findings [3, 11, 13, 14]. Most studies on NFL in SAH have been done using CSF, which leads to selection bias since they only include patients who are in need of an EVD and thus have hydrocephalus. As mentioned above, this only includes 1/3 of patients suffering from SAH [2]. The biomarker NFL corresponds to axonal injury [21] and could therefore be considered to be a surrogate marker for the amount of white matter tissue damage in SAH patients. Like previous groups, we observed a rise in sNFL levels over time. This seems to peak on day 7 [3, 4, 6]. At this timepoint, the risk of vasospasm/DCI is highest, presenting a potential explanation for the elevation of sNFL. Nylén et al. found a correlation between CSF-NFL levels and secondary ischemic events and presence of radiologic infarctions on follow-up CT [13]. We did not manage to reproduce those findings. However, our definitions of DCI and vasospasm were highly subjective compared with those in other studies, and interpretations involving these variables should be made with caution. We cannot, in this study, either dismiss or confirm the hypothesis that increasing NFL levels correlate with tissue damage caused by secondary events, such as DCI/vasospasm. One previous study did not show a correlation between NFL and initial WFNS scale score or outcome in SAH [21], but several others have [3, 4, 6]. We found a strong correlation between WFNS scale score upon admission and early sNFL values, probably because we did not have the selection bias of excluding patients who did not need an EVD.

Neurofilament levels in both serum and CSF have previously shown promising results in predicting outcome in SAH [3, 11]. We managed to reproduce these findings and observed that the late sNFL values had the highest predictive properties based on ROC curves. It is known that DCI/vasospasm contributes to unfavourable outcome [12]. Such events occur around this epoch—therefore, it is not surprising that the sNFL values at this time have the best predictive capability, since it most likely reflects a combination of initial tissue damage and tissue damage due to secondary events.

The ANOVA analysis showed that sNFL values significantly increased over time, and that there was a significant difference in sNFL values between the outcome groups in all epochs. This strengthens the hypothesis that the sNFL value reflects the amount of tissue damage caused by the initial bleed as well as secondary events. In our regression model, sNFL from the late phase showed significance only in the univariate analysis. When building a multivariate model, late phase sNFL lost its significance, contrary to the findings of Garland et al. [3]. This could probably be explained by the differences in our cohorts, since age and Fisher grade showed no correlation to outcome in that study, unlike in ours. However, the Nagelkerke pseudo *R*^2^ value increased when adding the late phase sNFL variable, which suggests increased predictive capability of the model. We do not believe WFNS scale score to be a confounder, but the strong correlation between WFNS scale score and sNFL suggests they both measure the amount of brain damage that has occurred. However, we cannot conclude that sNFL adds value to WFNS scale score when it comes to predicting outcome in SAH. A more extensive study with a larger number of samples and multiple clinical outcome measures is warranted to shed more light on the use of sNFL in predicting clinical outcome.

### Limitations of the study

The range in the timing of venous sampling is a factor to take into consideration when interpreting the results of this study. By dividing the samples into early, mid and late phase, we have partly accounted for this issue, and our results are similar to those of other studies [3]. We did not have a control group, which made it difficult to define normal levels of sNFL. This was partly managed by comparing levels with pre-specified cut-offs from a cohort in another Scandinavian country [5]. Vasospasm and DCI were assessed subjectively, and did not correlate with either outcome or sNFL levels, in contrast to previous data [12]. These variables should therefore be interpreted with caution, and their potential roles in explaining the rise in sNFL levels remain unclear.

## Conclusion

Measurements of sNFL with a highly sensitive ELISA method are precise and correlate with CSF-NFL levels. Serum NFL levels correlate with initial tissue damage measured clinically as WFNS scale score and with long-term clinical outcome measured as dichotomised GOSE score. Serum NFL levels rise over time, possibly reflecting secondary negative events. Further studies with frequent sampling and objective measurement of secondary events such as DCI and vasospasm are needed to explore potential mechanisms linking sNFL to these secondary events. Eventually, sNFL may be used in the clinical setting as a biomarker for the severity of the disease and in prognostication of clinical outcome.

### Supplementary information

ESM 1
