# Supplementary material for: Serum neurofilament light as a predictor of outcome in subarachnoid haemorrhage
Source: Acta Neurochir (Wien). 2023 Jun 23;165(10):2793–800. doi: 10.1007/s00701-023-05673-9 (PMC10542720; doi:10.1007/s00701-023-05673-9)
Supplement: Supplementary file 1 — ESM 1 [file 701_2023_5673_MOESM1_ESM.docx]

|  | N (Favourable/Unfavourable) |
| --- | --- |
| Day 0 | 60/25 |
| Day 1 | 13/2 |
| Day 2 | 11/4 |
| Day 3 | 29/10 |
| Day 4 | 8/6 |
| Day 5 | 13/2 |
| Day 6 | 21/6 |
| Day 7 | 25/12 |
| Day 8 | 12/2 |
| Day 9 | 7/2 |
| Day 10 | 24/8 |
| Day 11 | 4/4 |
| Day 12 | 9/2 |
| Day 13 | 8/2 |
| Day 14 | 8/10 |
| Day 15 | 0/0 |
| Day 16 | 0/0 |
| Day 17 | 2/0 |

**Supplemental material**

Number of serum samples available from each outcome group in relation to day from ictus. Favourable = Glasgow outcome scale extended score 5-8. Unfavourable = Glasgow outcome scale extended score 1-4.
